# Coherence resource power of isocoherent states

**DOI:** 10.1038/s41598-022-11300-x

**Published:** 2022-05-05

**Authors:** Marcelo Losada, Gustavo M. Bosyk, Hector Freytes, Giuseppe Sergioli

**Affiliations:** 1 FAMAF, CONICET, Universidad Nacional de Córdoba, Córdoba, Argentina; 2 UNLP, CONICET, Facultad de Ciencias Exactas, Instituto de Física La Plata, La Plata, Argentina; 3 Universitá degli Studi di Cagliari, Via Is Mirrionis 1, 09123 Cagliari, Italy

**Keywords:** Quantum information, Quantum mechanics, Qubits

## Abstract

We address the problem of comparing quantum states with the same amount of coherence in terms of their coherence resource power given by the preorder of incoherent operations. For any coherence measure, two states with null or maximum value of coherence are equivalent with respect to that preorder. This is no longer true for intermediate values of coherence when pure states of quantum systems with dimension greater than two are considered. In particular, we show that, for any value of coherence (except the extreme values, zero and the maximum), there are infinite incomparable pure states with that value of coherence. These results are not peculiarities of a given coherence measure, but common properties of every well-behaved coherence measure. Furthermore, we show that for qubit mixed states there exist coherence measures, such as the relative entropy of coherence, that admit incomparable isocoherent states.

## Introduction

Quantum coherence, which is a consequence of the superposition principle, is one of the fundamental aspects of the quantum theory. It has practical relevance in numerous fields of quantum physics, particularly in quantum information processing^1^. Furthermore, within the paradigm of quantum resource theories^2^, quantum coherence is considered as a quantum resource that can be converted, consumed and quantified^3,4^.

As any resource theory, the *resource theory of coherence* is built from three basic concepts: free states, resources and free operations. Since coherence is a basis-dependent notion, these three elements are defined in terms of a fixed basis, called *incoherent basis*. The free states of the theory, called *incoherent states*, are quantum states with diagonal density matrix in the incoherent basis. The rest of the states are resources and they are called *coherent states*. Regarding the free operations of the theory, there is no single definition and each proposal leads to a different resource theory for coherence, see *e.g.*^1^ and references therein. In this work, we follow the definition of an *incoherent operation* (IO) introduced in^3^, which has the property that coherence can not be created from an incoherent state, not even in a probabilistic way.

The resource-theoretic formulation allows us to introduce a *preorder* between quantum states induced by the incoherent operations: one state is more or equally coherent than other if the former can be converted into the later by means of incoherent operations. This preorder is useful for studying coherence transformations and classifying the set of quantum states according to its *coherence resource power*. Given any pair of quantum states, they can be classified as: (i) *IO-comparable*, when one state can be transformed into the other by means of IO, (ii) *IO-equivalent*, when both states can be transformed into the other, and (iii) *IO-incomparable*, when neither state can be transformed into the other.

Another way to capture operational aspects of coherence is by means of coherence quantifiers. There are several coherence quantifiers and most of them can be studied from an axiomatic point of view. More precisely, any *bonafide* coherence measure has to vanish only for incoherent states, to be strong monotone and convex^3^ and to be maximal for maximal coherent sates as discussed in^5^. Examples of coherence measures are the relative entropy of coherence^3^, the $\ell _1$-norm of coherence^3^ and the coherence of formation^6^, among others^7–13^.

Clearly, each coherence measure induces a total order on the quantum states. In general, these total orders are different. For instance, the total order induced by the relative entropy of coherence and by $\ell _1$-norm of coherence do not coincide^14^. This result motivates the comparison among other total orders induced by different coherence measures (see e.g.^15–20^).

In this work, we address a related but different problem concerning the ordering of quantum states with respect to coherence. In particular, we focus on quantum states with a fixed value of coherence and we ask for their coherence resource power in terms of the preorder induced by the incoherent operations. First, we observe that all quantum states with null coherence are IO-equivalent. Similarly, all quantum states with maximum coherence are also IO-equivalent. However, this is not true in general. In particular, in the case of pure states, we show that, for any value of coherence (except the extreme values, zero and the maximum), there are infinite IO-incomparable pure states with that value of coherence, provided that the dimension of the quantum systems is greater than two. These results are not peculiarities of a given coherence measure, but common properties of every well-behaved coherence measure. Furthermore, for qubit mixed states, we show that there are coherence measures, such as the relative entropy of coherence, that admit incomparable isocoherent states. In this way, our work complements other works^14–20^ about ordering of quantum states with coherence.

The paper is organized as follows. First, we review the main aspects of the resource theory of coherence, including the definition of incoherent states, incoherent operations and coherence measures, among other relevant results. Then, we provide our main results regarding the comparison of states with the same amount of coherence in terms of their coherence resource power. Finally, we summarize our results and discuss their roots and links with related problems. For the sake of readability all proofs and auxiliary results are given in the “Methods”.

## Preliminaries: resource theory of coherence, IO preorder and coherence measures

We will focus on quantum systems with finite dimension. The Hilbert space of the system is denoted by ${\mathscr {H}}$ and its dimension by $d = \dim {\mathscr {H}}$. The set of all quantum states of ${\mathscr {H}}$ is denoted by ${\mathscr {S}}({\mathscr {H}})$ and the subset of pure states is denoted by ${\mathscr {P}}({\mathscr {H}})$.

Without loss of generality we choose the computational basis ${\mathscr {B}}= \{{\vert }{i}{\rangle }\}^{d-1}_{i=0}$ as the incoherent basis. In this way, incoherent states, are quantum states with diagonal density matrix in the computational basis. More precisely, $\rho$ is an incoherent state if and only if $\rho = \sum ^{d-1}_{i=0} \lambda _i {\vert }{i}{\rangle }{\langle }{i}{\vert }$, with $\lambda _i \ge 0$ and $\sum _{i=0}^{d-1} \lambda _i =1$. We denote the set of incoherent states as ${\mathscr {I}}$.

The incoherent operations introduced in^3^, which have the property that coherence can not be created from an incoherent state, not even in a probabilistic way, are defined as follows. A completely positive trace-preserving map $\Lambda : {\mathscr {S}}({\mathscr {H}}) \rightarrow {\mathscr {S}}({\mathscr {H}})$ is an incoherent operation (IO) if it admits a representation in terms of Kraus operators $\{K_n\}^N_{n=1}$ such that $K_n \rho K^\dag _n/{\text {Tr}}(K_n \rho K^\dag _n) \in {\mathscr {I}}$ for all $1\le n \le N$ and $\rho \in {\mathscr {I}}$.

Interesting enough, incoherent operations induce a preorder among quantum states in terms of their coherence resource power:

### Definition 1

We say that the state $\rho$ *is more or equally coherent than* $\sigma$ if it is possible to transform the former into the latter by means of an incoherent operation. We denote this by $\rho \underset{\mathrm {IO}}{\rightarrow }\sigma$.

In other words, $\rho \underset{\mathrm {IO}}{\rightarrow }\sigma$ if there is an incoherent operation $\Lambda$ such that $\sigma = \Lambda (\rho )$. This defines a preorder on the set ${\mathscr {S}}({\mathscr {H}})$, since it satisfies the two conditions:

1. Reflexivity: $\rho \underset{\mathrm {IO}}{\rightarrow }\rho$ for all $\rho \in {\mathscr {S}}({\mathscr {H}})$.
2. Transitivity: If $\rho \underset{\mathrm {IO}}{\rightarrow }\omega$ and $\omega \underset{\mathrm {IO}}{\rightarrow }\sigma$, then $\rho \underset{\mathrm {IO}}{\rightarrow }\sigma$ for all $\rho ,\omega , \sigma \in {\mathscr {S}}({\mathscr {H}})$.

Property 1 follows from the fact that doing nothing, i.e., applying the identity operator, is an IO, whereas property 2 follows from the fact that the composition of IOs is an IO.

Furthermore, we classify the states as follows. We say that two states $\rho$ and $\sigma$ are *IO-comparable* when $\rho \underset{\mathrm {IO}}{\rightarrow }\sigma$ or $\sigma \underset{\mathrm {IO}}{\rightarrow }\rho$. In particular, if both of these transformations are possible, we say that they are *IO-equivalent*, and we denote that as $\rho \underset{\mathrm {IO}}{\leftrightarrow }\sigma$. On the contrary, if neither of these transformations are possible, we say that the states are *IO-incomparable*, and we denote that as $\rho \underset{\mathrm {IO}}{\nleftrightarrow }\sigma$.

We recall that, in this resource theory of coherence, there exist *maximally coherent states* (MCSs), which are states that can be transformed into any other state by means of IO. The canonical MCS is a pure state of the form $\rho ^{\text {mcs}} = {\vert }{\psi ^{\text {mcs}}_d}{\rangle }{\langle }{\psi ^{\text {mcs}}_d}{\vert }$^3^, with

1 $$\begin{aligned} {\vert }{\psi ^{\mathrm {mcs}}_d}{\rangle }= \frac{1}{\sqrt{d}} \sum _{i=0}^{d-1} {\vert }{i}{\rangle }. \end{aligned}$$

Any MCSs can be obtained from the state $\rho ^{\text {mcs}}$ by applying a unitary incoherent operations of the form $U_{\mathrm {IO}} = \sum _{i= 0}^{d-1} e^{\imath \theta _{i}} {\vert }{\pi (i)}{\rangle }{\langle }{i}{\vert }$, where $\pi$ is a permutation acting on the set $\{0,1,\ldots ,d-1\}$ and $\theta _{i} \in {\mathbb {R}}$^5^.

In addition, we recall that for pure states the preorder induced by IO is equivalent to the majorization preorder of the corresponding coherence vectors (see^7,8,21–23^). More precisely, given ${\vert }{\psi }{\rangle } \in {\mathscr {H}}$, its *coherence vector* is defined as the vector

2 $$\begin{aligned} \psi = \left( |{\langle }{0|\psi }{\rangle }|^2, \ldots , |{\langle }{d-1|\psi }{\rangle }|^2\right) . \end{aligned}$$

Notice that $\psi \in \Delta _d$, where $\Delta _d$ is the set of *d*-dimensional probability vectors, i.e., $\Delta _d = \{\psi =(\psi _0, \ldots ,\psi _{d-1}) \in {\mathbb {R}}^d: \psi _i \ge 0, \sum _{i= 0}^{d-1} \psi _i =1\}$.

Given two probability vectors $\psi =(\psi _0, \ldots ,\psi _{d-1})$ and $\phi =(\phi _0, \ldots ,\phi _{d-1})$, we say that $\psi$ is majorized by $\phi$, and we denote it by $\psi \preceq \phi$, if^24^

3 $$\begin{aligned} \sum _{i=0}^k \psi ^\downarrow _i \le \sum _{i=0}^k \phi ^\downarrow _i \quad \forall \, 0 \le k \le d-2, \end{aligned}$$

where $^\downarrow$ indicates that the entries of $\psi$ and $\phi$ are sorted in non-increasing order, i.e., $\psi ^\downarrow _i \ge \psi ^\downarrow _{i+1}$ and $\phi ^\downarrow _i \ge \phi ^\downarrow _{i+1}$ for all $0 \le i \le d-2$.

As we say before, the majorization relation defines a preorder on the set of probability vectors $\Delta _d$. If $\psi \preceq \phi$ or $\phi \preceq \psi$, we say that the probability vectors are *comparable*. If both relation are satisfied, $\psi$ and $\phi$ are equal up to a permutation. In this case we say that the probability vectors are *equivalent*. For dimensions greater than two^24^, there are cases in which neither $\psi \preceq \phi$ nor $\phi \preceq \psi$ are possible. When this is the case, we say that the probability vectors are *incomparable*.

Taking into account these definitions, we can state the following result that connects both preorders (see^7,8,21–23^).

### Theorem 1

Let ${\vert }{\psi }{\rangle }$ and ${\vert }{\phi }{\rangle }$ be two pure states. Then,

4 $$\begin{aligned} {\vert }{\psi }{\rangle } \underset{\mathrm {IO}}{\rightarrow }{\vert }{\phi }{\rangle } \iff \psi \preceq \phi . \end{aligned}$$

According to this theorem, two pure states ${\vert }{\psi }{\rangle }$ and ${\vert }{\phi }{\rangle }$ are IO-comparable (or IO-incomparable) if and only if their corresponding coherence vectors are comparable (or incomparable).

In general, for mixed states, a finite number of conditions are not sufficient to fully characterize coherent transformations^25^. From a generalized notion of coherence vector, it can be obtained a necessary condition in terms of a majorization relation^13^. However, qubit transformations under incoherent operations are completely characterized^26,27^.

### Theorem 2

Let $\rho$ and $\sigma$ be two qubit states with Bloch vectors $\mathbf {r} _1= (x_1,y_1,z_1)$ and $\mathbf {r} _2 = (x_2,y_2,z_2)$, respectively. Then, $\rho \underset{\mathrm {IO}}{\rightarrow }\sigma$ if and only if

5 $$\begin{aligned} r_1&\ge r_2, \end{aligned}$$

6 $$\begin{aligned} \frac{1- z^2_1}{r^2_1}&\le \frac{1- z^2_2}{r^2_2}, \end{aligned}$$

with $$r_1 = \sqrt{x^2_1 + y^2_1}$$ and $$r_2 = \sqrt{x^2_2 + y^2_2}$$.

In addition to the comparability notions between states, it is relevant to quantify the coherence amount of quantum states. In this paper, we mainly follow the axiomatic formulation for coherence measures.

### Definition 2

A coherence measure *C* is a real function defined on ${\mathscr {S}}({\mathscr {H}})$, satisfying the following conditions:

1. Vanishing only on incoherent states: $C\left( \rho \right) = 0$ if and only if $\rho \ \in {\mathscr {I}}$.
2. Strong monotonicity under IO: $C(\rho ) \ge \sum _i p_i C(\sigma _i)$, where $\{p_i,\sigma _i\}$ is an ensemble obtained from the state $\rho$ by means of IO.
3. Convexity: $$C\left( \sum _i p_i \rho _i \right) \le \sum _i p_i C(\rho _i)$$.
4. Maximum coherence: $\arg \max _{\rho \in {\mathscr {S}}({\mathscr {H}})} C(\rho )$ coincides with the set of maximally coherent states.

It can be shown that conditions 2 and 3 imply monotonicity under IO, that is, $C(\rho ) \ge C(\Lambda (\rho ))$ for any incoherent operation $\Lambda$ and any state $\rho$. The relevance of condition 4 is discussed in^5^. In particular, this condition excludes some problematic cases as the one given in Ex. 4 of^28^.

An interesting result is that any coherence measure restricted to pure states can be expressed in terms of a real, symmetric and concave functions defined on $\Delta _d$. More precisely, given the set

7 $$\begin{aligned} {\mathscr {F}}= \big \{f : \Delta _d \rightarrow [0,1]: f \ \text {is symmetric and concave,} \ f(1, 0, \ldots , 0) =0, \ \text {and} \ \arg \max _{\psi \in \Delta _d} f(\psi )= (1/d, \ldots , 1/d) \big \}, \end{aligned}$$

we have the following result^7,8^.

### Theorem 3

Let *C* be a coherence measure. Then, there exists a function $f_{C} \in {\mathscr {F}}$, such that the restriction of *C* to the set of pure states, denoted by $C|_{{\mathscr {P}}({\mathscr {H}})}$, satisfies

8 $$\begin{aligned} C|_{{\mathscr {P}}({\mathscr {H}})}({\vert }{\psi }{\rangle }{\langle }{\psi }{\vert }) = f_C \left( \psi \right) , \end{aligned}$$

where $\psi$ is the coherence vector of ${\vert }{\psi }{\rangle }$.

By abuse of notation, we will use $C({\vert }{\psi }{\rangle })$ when evaluating the restriction of the measure *C* on a pure state, instead of $C|_{{\mathscr {P}}({\mathscr {H}})}({\vert }{\psi }{\rangle }{\langle }{\psi }{\vert })$. In particular, we will focus on functions of ${\mathscr {F}}$ that are also strictly Schur-concave. Namely, a real function *f* defined on $\Delta _d$ is said to be *Schur-concave*, if $f(\psi ) \ge f(\phi )$ whenever $\psi \preceq \phi$. If, in addition, $f(\psi ) > f(\phi )$ whenever $\psi \preceq \phi$ and $\psi \ne \Pi \phi$, with $\Pi$ a permutation matrix, then *f* is said to be *strictly Schur-concave* (see Def. A.1 in^24^). We note that any function $f \in {\mathscr {F}}$ is Schur-concave, since they are symmetric and concave (see Prop. C.2 in^24^), but they are not necessarily strictly Schur-concave. The condition that $f_C$ be strictly Schur-concave is not so restrictive, since the most used coherence measures satisfy it, including the relative entropy of coherence, the $\ell _1$-norm of coherence, and the coherence of formation.

Now, we review some relevant coherence measures. The first one is the *relative entropy of coherence* $C_\mathrm {re}$, defined as

9 $$\begin{aligned} C_\mathrm {re}(\rho ) = \min _{\sigma \in {\mathscr {I}}} S(\rho ||\sigma ), \end{aligned}$$

where $S(\rho ||\sigma )= {\text {Tr}}\left( \rho (\ln \rho -\ln \sigma )\right)$. Alternatively, the relative of coherence can be expressed as $C_\mathrm {re}(\rho ) = S(\rho _{diag}) - S(\rho )$, where $\rho _{diag} = \sum ^{d-1}_{i=0} {\langle }{i|\rho |i}{\rangle } {\vert }{i}{\rangle }{\langle }{i}{\vert }$ and $S(\rho ) = -{\text {Tr}}(\rho \ln \rho )$ is the von Neumann entropy. Accordingly, the associated function $f_{C_\mathrm {re}} \in {\mathscr {F}}$ of the relative of coherence is the Shannon entropy, i.e., $f_{C_\mathrm {re}}(\psi ) = H(\psi )=- \sum _{i=0}^{d-1}\psi _i \ln \psi _i$, which is also strictly Schur-concave. The relative entropy of coherence has a particular operational interpretation. It coincides with the distillable coherence, that is, the maximal number of maximally coherent single-qubit states ${\vert }{\psi ^{\text {mcs}}_2}{\rangle }$ which can be obtained per copy of a given state $\rho$ by means of incoherent operations in the asymptotic limit^6^.

Another relevant coherence measure is given in terms of the off-diagonal elements of $\rho$. More precisely, the $\ell _1$-*norm of coherence* $C_{\ell _1}$ is defined as

10 $$\begin{aligned} C_{\ell _1}(\rho ) = \sum _{\begin{array}{c} i,i'=0 \\ i\ne i' \end{array}}^{d-1} |{\langle }{i|\rho |i'}{\rangle }|. \end{aligned}$$

In this case, the associated function $f_{C_{\ell _1}} \in {\mathscr {F}}$ is given by $f_{C_{\ell _1}}(\psi ) = \left( \sum _{i=0}^{d-1} \sqrt{\psi _i}\right) ^2 -1$, which is also strictly Schur-concave. We remark that the $\ell _1$-norm of coherence is useful for characterizing quantum interference and obtaining complementarity relations between coherence and path information in multipath interferometers^29–31^.

Finally, we recall that there are different ways to extend a coherence measure defined on pure states to mixed states^7,8,12,13^. The most common way is the convex roof method. For any $f \in {\mathscr {F}}$ the *convex roof measure of coherence* $C^{cr}_{f}$ is given by^7,8^

11 $$\begin{aligned} C^{cr}_{f}(\rho ) = \min _{\left\{ q_k, {\vert }{ \psi _k}{\rangle } \right\} _{k = 1}^M \in {\mathscr {D}}(\rho ) } \sum _{k=1}^M q_k f(\psi _k), \end{aligned}$$

where ${\mathscr {D}}(\rho )= \left\{ {\left\{ q_k, {\vert }{ \psi _k}{\rangle } \right\} }_{k = 1}^M : \ \rho = \sum _{k = 1}^{M} q_k {\vert }{\psi _k}{\rangle }{\langle }{\psi _k}{\vert } \right\}$ is set of all pure state decompositions of $\rho$. For instance, choosing the function $f \in {\mathscr {F}}$ as the *q*-Tsallis entropy^32^, i.e., $f^T_{q}(\psi ) = \left( 1- \sum _{i=0}^{d-1} \psi _i^q \right) /(q-1)$ for $q \in (0,1) \cup (1,+\infty )$, leads to the *q*-Tsallis coherence of formation $C^T_{q}$. Notice that $f^T_{q}$ is strictly Schur-concave^33^ and $C^T_{1}(\rho ) = \lim _{q \rightarrow 1} C^T_{q}(\rho ) = C_{CoF}(\rho )$, recovering the coherence of formation. This measure coincides with the coherence cost, that is, the minimal number of maximally coherent single-qubit states ${\vert }{\psi ^{\text {mcs}}_2}{\rangle }$ required to produce a given state $\rho$ by means of incoherent operations, in the asymptotic limit^6^.

## Results

We are interesting in comparing states with the same amount of coherence. For a given coherence measure *C* and a non-negative number $\alpha$, we introduce the set of isocoherence states ${\mathscr {E}}_{C,\alpha }$ as follows

12 $$\begin{aligned} {\mathscr {E}}_{C,\alpha } = \{\rho \in {\mathscr {S}}({\mathscr {H}}) : C(\rho ) = \alpha \}. \end{aligned}$$

On the one hand, from the condition 1 of definition 2, it follows ${\mathscr {E}}_{C,0} ={\mathscr {I}}$. Thus, as a consequence of that all incoherent states are IO-equivalent (see Observation 1), the states belonging to ${\mathscr {E}}_{C,0}$ are all IO-equivalent. On the other hand, from condition 4 of definition 2, it follows that the set ${\mathscr {E}}_{C,M_C}$, with $M_C = C(\rho ^{\text {mcs}})$, is formed by maximally coherent states. Thus, the states belonging to ${\mathscr {E}}_{C,M_C}$ are all IO-equivalent. Therefore, as one might expect, all isocoherent states with an extreme value of coherence have the same coherence resource power. Moreover, this is also true for pure isocoherent states of qubit systems for any value of coherence.

### Proposition 1

For any function $f \in {\mathscr {F}}$ strictly Schur-concave, pure isocoherent states of qubit systems are IO-equivalent.

A natural question that arises from the previous observations is: In higher dimensional systems, do isocoherent pure states with an intermediate value of coherence have the same coherence resource power? In other words, for systems with $d>2$, we are asking if states of ${\mathscr {E}}_{C,\alpha }$, with $\alpha \in (0,M_C)$, are IO-equivalent.

We will show that this is not the case. More precisely, we will prove that for each value of coherence $\alpha$ in the interval $(I_{C}, M_{C})$, there are infinite IO-incomparable pure states with that amount of coherence, where $I_{C}= \inf _{\psi \in \text {ri}(\Delta _d)} f_{C}(\psi )$ with $\text {ri}(\Delta _d)$ the relative interior of the set $\Delta _d$, i.e., $\text {ri}(\Delta _d) = \{\psi \in {\mathbb {R}}^d: \psi _i > 0, \sum _{i= 0}^{d-1} \psi _i =1\}$.

### Proposition 2

Let $d>2$ and let $C : {\mathscr {S}}({\mathscr {H}}) \rightarrow {\mathbb {R}}$ be a coherence measure such that its restriction to pure states has an associated function $f_C \in {\mathscr {F}}$ strictly Schur-concave. For any $\alpha \in (I_{C}, M_{C})$, there are infinite pure states $\{{\vert }{\psi ^{(i)}}{\rangle }{\langle }{\psi ^{(i)}}{\vert }\}_{i \in {I}} \subseteq {\mathscr {E}}_{C,\alpha }$ (with ${I}$ a set of index), such that ${\vert }{\psi ^{(i)}}{\rangle } \underset{\mathrm {IO}}{\nleftrightarrow }{\vert }{\psi ^{(i')}}{\rangle }$ for all $i \ne i' \in {I}$.

Another condition that coherence measures usually satisfy is the continuity condition, that is

$$5.$$
Continuity: *C* is continuous on ${\mathscr {S}}({\mathscr {H}})$.

If a coherence measure also satisfies the continuity condition ($5$), we have that for any possible value of coherence (except the extreme cases zero and the maximal value $M_C$) there are an infinite number of IO-incomparable pure states with that amount of coherence.

### Corollary 1

Let $d>2$ and let $C : {\mathscr {S}}({\mathscr {H}}) \rightarrow {\mathbb {R}}$ be a coherence measure satisfying condition ($5$) and such that its restriction to pure states has an associated function $f_C \in {\mathscr {F}}$ strictly Schur-concave. For any $\alpha \in (0, M_{C})$, there are infinite pure states $\{{\vert }{\psi ^{(i)}}{\rangle }{\langle }{\psi ^{(i)}}{\vert }\}_{i \in {I}} \subseteq {\mathscr {E}}_{C,\alpha }$ (with ${I}$ a set of index), such that ${\vert }{\psi ^{(i)}}{\rangle } \underset{\mathrm {IO}}{\nleftrightarrow }{\vert }{\psi ^{(i')}}{\rangle }$ for all $i \ne i' \in {I}$.

In particular, we remark that, Propositions 1 and 2, and Corollary 1 are valid for the relative entropy of coherence $C_\mathrm {re}$ and the $\ell _1$-norm of coherence $C_{\ell _1}$.

Finally, for the three-dimensional case, we provide an example of a family of IO-incomparable pure states with the same value of the relative entropy of coherence. For each $a \in [0,1]$, we define the curve

13 $$\begin{aligned} {\left\{ \begin{array}{ll} \psi ^{(a)}: [0,1] \rightarrow \Delta ^\downarrow _d \\ \psi ^{(a)}(t) = u + t (v - u) + a t (t-1) w, \end{array}\right. } \end{aligned}$$

where $u=(1/3,1/3,1/3)$, $v=(1,0,0)$ and $w=(0,-1/3,1/3)$. For each $a\in [0,1]$ and $t \in [0,1]$, $\psi ^{(a)}(t)$ is a probability vector sorted in a non-increasing way. Moreover, it can be proved that different curves do not have equivalent probability vectors in common, except the extreme vectors *u* and *v*.

**Figure 1:**
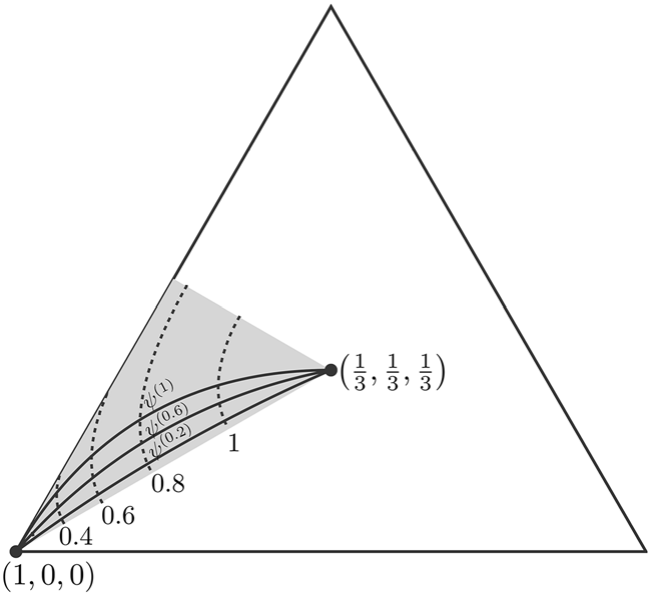
The big triangle is the set of three-dimensional probability vectors $\Delta _3$. The gray triangle is the subset of ordered probability vectors $\Delta ^\downarrow _3$. We depict the curves $\psi ^{(a)}$, defined in Eq. (13), for $a \in \{0.2,0.6,1\}$ (solid lines) and the contours plot of $-\sum ^2_{i=0} \psi _i \ln \psi _i = \alpha$ for $\alpha \in \{0.2,0.4,0.8,1\}$ (dashed lines). The intersection of a given contour plot with the curves $\psi ^{(a)}$ gives a family of incomparable probability vectors. From this family and Eq. (14), we obtain a family of mutually IO-incomparable pure states with relative entropy of coherence equal to $\alpha$.

For some $\alpha \in (0, M_C)$ (with $M_C=\ln 3$) and for each $a \in [0,1]$, we consider the intersection of the contour plot $C_{r}({\vert }{\psi }{\rangle })= - \sum ^2_{i=0} \psi _i \ln \psi _i = \alpha$ with the curve $\psi ^{(a)}$. We denote this intersection by $\psi ^{(a)}(t_a^*)$, with $t_a^* \in (0,1)$. In Fig. 1, we depict the curves $\psi ^{(a)}$, for $a \in \{0.2,0.6,1\}$ (solid lines), and the contour plots $C_{r}({\vert }{\psi }{\rangle }) = \alpha$, for $\alpha \in \{0.2,0.4,0.6,0.8,1\}$ (dashed lines).

We consider the family of probability vectors $\{\psi ^{(a)}(t_a^*)\}_{a \in [0,1]}$, and the corresponding family of pure states $\{{\vert }{\psi ^{(a)}}{\rangle }\}_{a \in [0,1]}$, where

14 $$\begin{aligned} {\vert }{\psi ^{(a)}}{\rangle } =\sum _{i=0}^{d-1} \sqrt{\left( \psi ^{(a)}(t^*_a)\right) _i} {\vert }{i}{\rangle }. \end{aligned}$$

Then, we have $C_{r}({\vert }{\psi ^{(a)}}{\rangle }) =f_{C_{r}}(\psi ^{(a)}(t_a^*)) = \alpha$ for all the states of the family. Since Shannon entropy is strictly Schur-concave, then the family of probability vectors only has equivalent or incomparable pairs of vectors. Moreover, since different curves do not have pairs of equivalent vectors (except the extreme vectors), the family of probability vectors does not have pairs of equivalent vectors. This implies that all probability vectors of the family $\{\psi ^{(a)}(t_a^*)\}_{a \in [0,1]}$ are mutually incomparable. We conclude that all the states of the family $\{{\vert }{\psi ^{(a)}}{\rangle }\}_{a \in [0,1]}$ are mutually IO-incomparable. In this way, we have found a family of mutually IO-incomparable pure states with relative entropy of coherence equal to $\alpha$.

Regarding qubit mixed states, we can distinguish two situations depending on the coherence measure. An arbitrary coherence measure for a qubit state $\rho$, with Bloch vector $\mathbf {r} = (x,y,z)$, can be expressed in terms of $r =\sqrt{x^2+y^2}$ and *z*, i.e., $C(\rho ) = C(r, z)$. For measures that do not depend on *z* and are strictly increasing on *r*, it is easy to see that there are no pair of incomparable isocoherent states. More precisely, let $\rho$ and $\sigma$ be two isocoherent states, with Bloch vectors $\mathbf {r}_1 = (x_1,y_1,z_1)$ and $\mathbf {r}_2= (x_2,y_2,z_2)$ respectively. Then, since the coherence measure only depends on *r*, and it is strictly increasing, we have that $r_1 = r_2$. Finally, from Theorem 2, we conclude that, if $|z_1| \le |z_2|$, then $\sigma \underset{\mathrm {IO}}{\rightarrow }\rho$, and if $|z_2| \le |z_1|$, then $\rho \underset{\mathrm {IO}}{\rightarrow }\sigma$. In other words, the isocoherent states $\rho$ and $\sigma$ are IO-comparable . In particular, the $\ell _1$-norm of coherence is an example of this kind of coherence measures: $C_{\ell _1}(\rho ) = r$.

**Figure 2:**
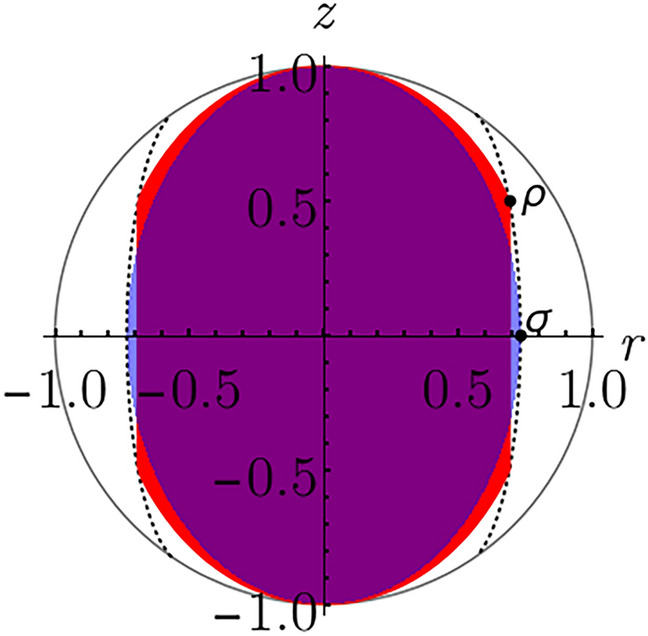
Projection of the Bloch sphere on the $z-r$ plane. The black dots represent two qubit states: $\rho$ (upper dot, with $r_1=0.69135, ~ z_1=0.5$) and $\sigma$ (lower dot, with $r_2= 0.732828, ~ z_2 = 0$). The red and blue regions represent the projection of the sets $\{\rho ': \rho \underset{\mathrm {IO}}{\rightarrow }\rho ' \}$ and $\{\rho ': \sigma \underset{\mathrm {IO}}{\rightarrow }\rho ' \}$ on the $z-r$ plane, respectively. The dotted black curve represents the set $\{\rho ': C_\mathrm {re}(\rho ') = 0.3\}$ projected on the $z-r$ plane. From the figure, it can be observed that $\rho$ and $\sigma$ are IO-incomparable, and $C_\mathrm {re}(\rho )= C_\mathrm {re}(\sigma )=0.3$.

For coherence measures that also depend on *z*, there are examples of isocoherent states which are incomparable. For instance, for the relative entropy of coherence, which can be expressed as $C_\mathrm {re}(\rho ) = h\left( \frac{1+z}{2} \right) - h\left( \frac{1+\sqrt{r^2+z^2}}{2} \right)$ with $h(x) = -x \ln x -(1-x) \ln (1-x)$, we show in Fig. 2 an example of two incomparable and isocoherent states.

## Discussions

In this work, we have considered the subset of quantum states formed by those states with a fixed value of coherence for a given coherence measure. We have analyzed its coherence resource power in terms of the preorder induced by the incoherent operations.

First, we have observed that, as one might expect, isocoherent states with an extreme value of coherence have the same coherence resource power in terms of the incoherent preorder. Second, we have shown that pure isocoherent states of qubit systems with arbitrary value of coherence are IO-equivalent (Proposition 1).

Third, we have proved that, in higher dimensional systems ($d>2$), pure isocoherent states are not necessarily IO-equivalent. In particular, for any value of coherence, we have shown that there are infinite IO-incomparable pure states with that value of coherence (Proposition 2 and Corollary 1). The essence of these results is that, in general, the coherence measures do not fully preserve the preorder structure of the quantum states induced by incoherent operations. Indeed, coherence measures map the set of quantum states to the positive real numbers, which is a total order set. In this way, the quantum states go from being pre-ordered by IO to being totally ordered by the coherence measure. Our results Proposition 2 and Corollary 1 arise as a consequence of this discrepancy. Another related consequence is the fact that different coherence measures induce different total orders on the set of quantum states, as it was observed in^14,16–18^. In this way, our work complements these studies about ordering quantum states with coherence.

Regarding qubit mixed states, we have distinguished two situations depending on the coherence measure. For measures that do not depend on *z* and are strictly increasing on *r*, we have shown that there are no pair of incomparable isocoherent states. In particular, the $\ell _1$-norm of coherence is an example of this kind of coherence measures. For coherence measures that also depend on *z*, we have shown that there are examples of isocoherent mixed states which are incomparable. In particular, we have considered the case of the relative entropy of coherence.

Finally, we remark that we have focused on the resource theory of coherence to illustrate these observations due to its topicality and practical relevance^1^. However, as our proofs are posed in a wider and simpler context based on majorization theory^24^, the results can be easily extended to any majorization-based quantum resource theory^34^, such as entanglement theory^35^ and nonuniformity^36,37^. In fact, the observations made for the entanglement entropy in^38^, namely there are infinite incomparable bipartite pure states with a fixed value of entanglement entropy, can be extend to any entanglement monotone^39^ by exploiting similar majorization arguments to those given in our proofs. The reason behind this generality is again that the preorder induced by the free operations of the resource theory and the total order induced by the measures are not isomorphic.

## Methods

First, we provide a proof of the following intuitive result: all incoherent states have the same coherence resource power. Indeed, for any quantum resource theory that admits a tensor product structure, like the quantum coherence resource theory considered here, it is valid that the free states are interconvertible by means of the free operations of the theory^2^. For the sake of completeness, we provide a directly proof of the Observation 1, giving an explicit incoherent operation that allows to transform an arbitrary incoherent state into another arbitrary one.

### Observation 1

All incoherent states are IO-equivalent.

### Proof

Let us prove that any incoherent state $\rho$ is IO-equivalent to the state ${\vert }{0}{\rangle }{\langle }{0}{\vert }$. By definition, $\rho = \sum ^{d-1}_{i=0} \lambda _i {\vert }{i}{\rangle }{\langle }{i}{\vert }$, with $\lambda _i \ge 0$ and $\sum _{i=0}^{d-1} \lambda _i =1$.

Firstly, let us consider the quantum operation $\Lambda _1$ with Kraus operators $K_i= {\vert }{0}{\rangle }{\langle }{i}{\vert }$, for $i = 0,\ldots , d-1$. It is easy to see that $\Lambda _1$ is an incoherent operation. Moreover, $\Lambda _1(\rho ) = {\vert }{0}{\rangle }{\langle }{0}{\vert }$. Therefore, for all $\rho \in {\mathscr {I}}$, $\rho \underset{\mathrm {IO}}{\rightarrow }{\vert }{0}{\rangle }{\langle }{0}{\vert }$.

Secondly, let define the quantum operation $\Lambda _2$ with Kraus operators $K_i= \sqrt{\lambda _i}{\vert }{i}{\rangle }{\langle }{0}{\vert }$, for $i = 0,\ldots , d-1$ and $G = I- {\vert }{0}{\rangle }{\langle }{0}{\vert }$. Again, it is easy to see that $\Lambda _2$ is an incoherent operation, and $\Lambda _2({\vert }{0}{\rangle }{\langle }{0}{\vert }) = \rho$. Then, for all $\rho \in {\mathscr {I}}$, ${\vert }{0}{\rangle }{\langle }{0}{\vert } \underset{\mathrm {IO}}{\rightarrow }\rho$.

Both results imply that, for all $\rho \in {\mathscr {I}}$, ${\vert }{0}{\rangle }{\langle }{0}{\vert } \underset{\mathrm {IO}}{\leftrightarrow }\rho$. Then, we can conclude that all incoherent states are IO-equivalent.

$$\square$$

Proof of Proposition 1

### Proof

Let ${\vert }{\psi }{\rangle }$ and ${\vert }{\phi }{\rangle }$ be two pure isocoherent states of a qubit system, and let $\psi ^\downarrow =(\psi ^\downarrow _0, 1- \psi ^\downarrow _0)$ and $\phi ^\downarrow =(\phi ^\downarrow _0, 1- \phi ^\downarrow _0)$ be their ordered coherence vectors.

In this case, Theorem 1 reduces to ${\vert }{\psi }{\rangle } \underset{\mathrm {IO}}{\rightarrow }{\vert }{\phi }{\rangle } \iff \psi ^\downarrow _0 \le \phi ^\downarrow _0$. Therefore, $\psi$ and $\phi$ are comparable or, equivalently, ${\vert }{\psi }{\rangle }$ and ${\vert }{\phi }{\rangle }$ are IO-comparable.

Moreover, since $f_C(\psi ) = f_C(\phi )$ and $f_C$ is strictly Schur-concave, we have that $\psi$ and $\phi$ are equivalent. Therefore, ${\vert }{\psi }{\rangle }$ and ${\vert }{\phi }{\rangle }$ are IO-equivalent.

$$\square$$

For the proof of Proposition 2 we need the following lemma.

### Lemma 1

Let $C : {\mathscr {S}}({\mathscr {H}}) \rightarrow {\mathbb {R}}$ be an coherence measure and $f_{C}$ its associated function.

(i) $f_{C}$ is continuous on $\text {ri}(\Delta _d)$, where $\text {ri}(\Delta_d)$ is the relative interior of $\Delta _d$, i.e., $\text {ri}(\Delta _d) = \{\psi \in {\mathbb {R}}^d: \psi _i > 0, \sum _{i= 0}^{d-1} \psi _i =1\}$,
(ii) If *C* satisfies condition ($5$), then $f_{C}$ is continuous on $\Delta _d$.
(iii) $I_{C} \equiv \inf _{\psi \in \text {ri}(\Delta _d)} f_{C}(\psi ) = \lim _{n \rightarrow \infty } f_{C}(v_n)$, with $v_{n} = (1- 1/n, 1/[n(d-1)], \ldots ,1/[n(d-1)])$. In particular, if $f_{C}$ is continuous on $\Delta _d$, $I_{C} = 0$.

### Proof

(i) Since $f_C$ is a concave function on $\Delta _d$, $-f_C$ is a convex function on the same domain. We consider the following extension of $f_C$ over all ${\mathbb {R}}^d$:
  $$\begin{aligned} g_C(\psi ) = \left\{ \begin{array}{ll} \!\! -f_C(\psi ) &{}~\text {if}~~ \psi \in \Delta _d \\ ~\!\! +\infty &{}~\text {if}~~ \psi \notin \Delta _d \end{array} \right. \end{aligned}$$
  The function $g_C$ is convex on ${\mathbb {R}}^d$, therefore it is continuous on $\text {ri}(\text {dom} \, g)$ (see Th. 10.1 in^40^). Since $\text {ri}(\text {dom} \, g_C) = \text {ri} (\Delta _d)$, $g_C$ is continuous on $\text {ri} (\Delta _d)$. Finally, we conclude that $f_C$ is continuous on $\text {ri} (\Delta _d)$.
(ii) We consider the continuous map $h: \Delta _d \mapsto {\mathscr {H}}$, given by
  15 $$\begin{aligned} h(\psi ) = \sum _{i=0}^{d-1}\sqrt{\psi _i} {\vert }{i}{\rangle }. \end{aligned}$$
  We can express the function $f_{C}$ in terms of *C* and *h* as follows,
  $$\begin{aligned} f_{C}(\psi ) = C(h(\psi )). \end{aligned}$$
  Therefore, if *C* is continuous, then $f_{C}$ is also continuous.
(iii) The probability vectors $v_{n} = (1- 1/n, 1/[n(d-1)], \ldots ,1/[n(d-1)])$ satisfy $v_n \prec v_{n+1}$ for all $n > 1$. Then, since $f_{C}$ is a bounded Schur-concave function, $\{f_{C}(v_n)\}_{n\in {\mathbb {N}}_{>1}}$ is a bounded and monotonic decreasing sequence. Therefore, there exists $L = \lim _{n \rightarrow \infty } f_{C}(v_n)$, and $L \le f_{C}(v_n)$ for all $n > 1$.
  Let $\psi \in \text {ri}(\Delta _d)$. Since $\lim _{n \rightarrow \infty } v_n = (1, 0, \ldots , 0)$, we have that there is some $n_\psi \in {\mathbb {N}}$, such that $\psi \preceq v_{n_\psi }$. Then, $f_{C}(\psi ) \ge f_{C}(v_{n_\psi }) \ge L$. Therefore, for all $\psi \in \text {ri}(\Delta _d)$, $f_{C}(\psi ) \ge L$, which implies $I_{C} = \inf _{\psi \in \text {ri}(\Delta _d)} f_{C}(\psi ) \ge L$.
  On the other hand, for all $\psi \in \text {ri}(\Delta _d)$, $I_{C} \le f_{C}(\psi )$. In particular, $I_{C} \le f_{C}(v_n)$ for all $n\in {\mathbb {N}}$. This implies, $I_{C} \le \lim _{n \rightarrow \infty } f_{C}(v_n)$.
  Summing up, $I_{C} = \lim _{n \rightarrow \infty } f_{C}(v_n)$. In particular, if $f_{C}$ is continuous on $\Delta _d$, we have $I_{C} = \lim _{n \rightarrow \infty } f_{C}(v_n) = f_{C} (1,0, \ldots , 0) = 0$.

$$\square$$

Proof of Proposition 2.

### Proof

On the one hand, $M_C = C(\rho ^{\text {mcs}}) = f_C(u)$, with $u = (1/d, \ldots , 1/d)$. On the other hand, due to Lemma 1, $I_C = \lim _{n \rightarrow \infty } f_{C}(v_n)$. Since, for all $n \in {\mathbb {N}}$, $u \preceq v_n$, but $v_n \npreceq u$, and $f_C$ is strictly Schur-concave, we have $f_C(v_n) < f_C (u)$. Therefore, $I_C < M_C$.

Let $\alpha \in (I_C, M_C)$. Since $\alpha > I_C = \lim _{n \rightarrow \infty } f_{C}(v_n)$ and $\{f_{C}(v_n)\}_{n\in {\mathbb {N}}_{>1}}$ is monotonic decreasing, there is some $n_{\alpha } >1$, such that $f_{C}(v_{n_{\alpha }}) < c$. Therefore, $f_{C}(v_{n_{\alpha }})< \alpha <f_{C}(u)$.

Now, we construct a family of probability vectors, such that the value of $f_{C}$ on these vectors is equal to $\alpha$. For each $a \in [0,1/d]$, we define the curve

$$\begin{aligned} \psi ^{(a)}(t) = u + t (v_{n_{\alpha }} - u) + a t (t-1) w ~~ \text { for } ~~ t \in [0,1], \end{aligned}$$

with $$v_{n_{\alpha }} = (1- 1/n_{\alpha }, 1/[n_{\alpha }(d-1)], \ldots ,1/[n_{\alpha }(d-1)])$$ and $$w= (0, -1/d, \ldots , -1/d, (d-2)/d)$$.

Notice that the entries of $\psi ^{(a)}(t)$ are

$$\begin{aligned} \psi ^{(a)}_1(t)&= \frac{(1-t)}{d}+ \frac{t (n_{\alpha } -1 )}{n_{\alpha }}, \\ \psi ^{(a)}_i(t)&= \frac{(1-t)}{d}+ \frac{t}{n_{\alpha }(d-1)} + \frac{a t(1-t)}{d}, ~ \text { for } ~ 2 \le i < d, \\ \psi ^{(a)}_{d}(t)&= \frac{(1-t)}{d}+ \frac{t}{n_{\alpha }(d-1)} - \frac{a t(1-t)(d-2)}{d}. \end{aligned}$$

Then, for $t \in [0,1]$ and $a \in [0,1/d]$, all the entries are greater than zero, and $\sum _{i=0}^{d-1} \psi ^{(a)}_i(t) =1$. In other words, for each $a \in [0,1/d]$, the curve $\psi ^{(a)}(t)$ is formed by probability vectors. In addition, $\psi ^{(a)}(t) \in \text {ri} (\Delta _d)$ for $t \in [0,1]$ and $a \in [0,1/d]$.

Next, we show that different curves do not have equivalent probability vectors in common, except *u* and $v_{n_{\alpha }}$. First, we observe that their entries are decreasingly ordered:

$$\begin{aligned} \psi ^{(a)}_1(t) - \psi ^{(a)}_2(t)&=t - \frac{t d}{n_{\alpha }(d-1)} - \frac{a t(1-t)}{d} \ge 0, \\ \psi ^{(a)}_{i}(t) - \psi ^{(a)}_{i+1}(t)&= 0, ~ \text { for } ~ 2 \le i < d-1, \\ \psi ^{(a)}_{d-1}(t) -\psi ^{(a)}_d(t)&= \frac{a t (1-t) (d-1)}{d} \ge 0. \end{aligned}$$

This implies that two probability vectors $\psi ^{(a)}(t)$ and $\psi ^{(a')}(t')$ are equivalent if, and only if, their entries are the same. There are three cases: (i) $t = t'=0$, (ii) $t=t' = 1$ or (iii) $t= t'$ and $a=a'$. Therefore, different curves do not have equivalent probability vectors in common, except *u* and $v_{n_c}$.

Finally, we construct a family of IO-incomparable pure states with amount of coherence equal to *c*. From Lemma 1, we have that $f_{C}$ is continuous on $\text {ri} (\Delta _d)$. Moreover, $\psi ^{(a)}(t) \in \text {ri} (\Delta _d)$ for $t \in [0,1]$ and $a \in [0,1/d]$. Therefore, for each $a \in [0,1/d]$, $f_{C}(\psi ^{(a)}(t))$ is a continuous function of the variable $t \in \left[ 0, 1\right]$. Since $f_{C}(\psi ^{(a)}(0)) = f_{C}(u)$, $f_{C}(\psi ^{(a)}(1)) =f_{C}(v_{n_c})$ and $f_{C}(v_{n_c})< c <f_{C}(u)$, there is a $t_a^*\in \left( 0, 1 \right)$ such that $f_{C}(\psi ^{(a)}(t_a^*))=c$. From the probability vectors $\psi ^{(a)}(t_a^*)$, we define the pure states ${\vert }{\psi ^{(a)}}{\rangle } = h(\psi ^{(a)}(t_a^*))$, with map *h* as in Eq. (15).

All pure states of the family $\{{\vert }{\psi ^{(a)}}{\rangle }\}_{a \in [0,1/d]}$ satisfy $C ({\vert }{\psi ^{(a)}}{\rangle })=\alpha$. Moreover, since the probability vectors of the family $\{\psi ^{(a)}(t_a^*)\}_{a \in [0,1/d]}$ are not mutually equivalent, then their respective states are not mutually IO-equivalent. Now, let us prove that they are IO-incomparable. Given $a, a' \in [0,1/d]$, suppose that ${\vert }{\psi ^{(a)}}{\rangle } \underset{\mathrm {IO}}{\rightarrow }{\vert }{\psi ^{(a')}}{\rangle }$. Then, ${\vert }{\psi ^{(a')}}{\rangle } \underset{\mathrm {IO}}{\nrightarrow }{\vert }{\psi ^{(a)}}{\rangle }$. In terms of the probability vectors this implies that $\psi ^{(a')} \preceq \psi ^{(a)}$, but $\psi ^{(a)} \npreceq \psi ^{(a')}$. Then, $\alpha = f_C(\psi ^{(a)}) < f_C(\psi ^{(a')})= \alpha$, which is absurd. Therefore, the pure states of the family $\{{\vert }{\psi ^{(a)}}{\rangle }\}_{a \in [0,1/d]}$ are mutually IO-incomparable. $\square$

Proof of Corollary 1.

### Proof

This result follows from Lemma 1 and Proposition 2. $\square$

## References

[1] Streltsov A, Adesso G, Plenio MB (2017). Colloquium: quantum coherence as a resource. Rev. Mod. Phys..

[2] Chitambar E, Gour G (2019). Quantum resource theories. Rev. Mod. Phys..

[3] Baumgratz T, Cramer M, Plenio MB (2014). Quantifying Coherence. Phys. Rev. Lett..

[4] Åberg, J. Quantifying Superposition, arXiv:quant-ph/0612146.

[5] Peng Y, Jiang Y, Fan H (2016). Maximally coherent states and coherence-preserving operations. Phys. Rev. A.

[6] Winter A, Yang D (2016). Operational resource theory of coherence. Phys. Rev. Lett..

[7] Du S, Bai Z, Qi X (2015). Coherence measures and optimal conversion for coherent states. Quant. Inf. Comp. A.

[8] Zhu H, Ma Z, Zhu C, Fei S, Vedral V (2017). Operational one-to-one mapping between coherence and entanglement measures. Phys. Rev. A.

[9] Rastegin AE (2016). Quantum-coherence quantifiers based on the Tsallis relative $$\alpha$$ entropies. Phys. Rev. A.

[10] Feng XN, Wei LF (2017). Quantifying quantum coherence with quantum Fisher information. Sci. Rep..

[11] Muthuganesana R, Chandrasekara VK, Sankaranarayananb R (2021). Quantum coherence measure based on affinity. Phys. Lett. A.

[12] Yu D, Zhang L, Yu C (2020). Quantifying coherence in terms of the pure-state coherence. Phys. Rev. A.

[13] Bosyk GM, Losada M, Massri C, Freytes H, Sergioli G (2021). Generalized coherence vector applied to coherence transformations and quantifiers. Phys. Rev. A.

[14] Liu CL, Yu XD, Xu GF, Tong DM (2016). Ordering states with coherence measures. Quantum Inf. Process..

[15] Zhang FG, Shao LH, Luo Y, Li Y (2016). Ordering states with Tsallis relative $$\alpha$$-entropies of coherence. Quantum Inf. Process..

[16] Yang LM, Chen B, Fei SM, Wang ZX (2018). Tong, Ordering states with various coherence measures. Quantum Inf. Process..

[17] Zhang FG, Li YM (2018). Sufficient Conditions of the Same State Order Induced by Coherence. Commun. Theor. Phys..

[18] Zhang J, Sheng YH, Tao YH, Fei SM (2021). Ordering states of $$l_1$$ norm and $$\alpha$$-affinity of coherence. Quantum Inf. Process..

[19] Mishra S, Thapliyal K, Pathak A, Venugopalan A (2019). Comparing coherence measures for X states: can quantum states be ordered based on quantum coherence?. Quantum Inf. Process..

[20] Mishra S, Thapliyal K, Pathak A (2022). Attainable and usable coherence in X states over Markovian and non-Markovian channels. Quantum Inf. Process..

[21] Du S, Bai Z, Guo Y (2015). Conditions for coherence transformations under incoherent operations. Phys. Rev. A.

[22] Du S, Bai SZ, Guo Y (2017). Erratum: conditions for coherence transformations under incoherent operations [Phys. Rev. A 91, 052120 (2015)],. Phys. Rev. A.

[23] Chitambar E, Gilad G (2016). Conditions for coherence transformations under incoherent operations. Phys. Rev. A.

[24] Marshal AW, Olkin I, Arnold BC (2011). Inequalities: theory of majorization and its applications.

[25] Du S, Bai Z, Qi X (2019). Coherence manipulation under incoherent operations. Phys. Rev. A.

[26] Streltsov A, Rana S, Boes P, Eisert J (2017). Structure of the resource theory of quantum coherence. Phys. Rev. Lett..

[27] Shi HL, Wang XH, Liu SY, Yang WL, Yang ZY, Fan H (2017). Coherence transformations in single qubit systems. Sci. Rep..

[28] Bai Z, Du S (2015). Maximally coherent states. Quantum Inf. Comput..

[29] Bera MN, Qureshi T, Siddiqui MA, Pati AK (2015). Duality of quantum coherence and path distinguishability. Phys. Rev. A.

[30] Bagan E, Bergou JA, Cottrell SS, Hillery M (2016). Relations between Coherence and Path Information. Phys. Rev. Lett..

[31] Mishra S, Venugopalan A, Qureshi T (2019). Decoherence and visibility enhancement in multipath interference. Phys. Rev. A.

[32] Tsallis C (1988). Possible generalization of Boltzmann-Gibbs statistics. J. Stat. Phys..

[33] Bosyk GM, Zozor S, Holik F, Portesi M, Lamberti PW (2016). A family of generalized quantum entropies: definition and properties. Quantum Inf. Process..

[34] Bosyk GM, Bellomo G, Holik F, Freytes H, Sergioli G (2019). Optimal common resource in majorization-based resource theories. New J. Phys..

[35] Nielsen MA (1999). Conditions for a class of entanglement transformations. Phys. Rev. Lett..

[36] Gour G, Müller NP, Narasimhachar V, Spekkens RW, Halpern NY (2015). The resource theory of informational nonequilibrium in thermodynamic. Phys. Rep..

[37] Streltsov A, Kampermann H, Wölk S, Gessner M, Bruß D (2018). Maximal coherence and the resource theory of purity. New J. Phys..

[38] Chattopadhyay I, Sarkar D (2008). Character of locally inequivalent classes of states and entropy of entanglement. Phys. Rev. A.

[39] Vidal G (2000). Entanglement monotones. J. Mod. Opt..

[40] Rockafellar RT (1970). Convex analysis.

